# Sex‐specific role of the circadian transcription factor NPAS2 in opioid tolerance, withdrawal and analgesia

**DOI:** 10.1111/gbb.12829

**Published:** 2022-08-20

**Authors:** Stephanie Puig, Micah A. Shelton, Kelly Barko, Marianne L. Seney, Ryan W. Logan

**Affiliations:** ^1^ Department of Pharmacology and Experimental Therapeutics Boston University School of Medicine Boston Massachusetts USA; ^2^ Translational Neuroscience Program, Department of Psychiatry University of Pittsburgh School of Medicine Pittsburgh Pennsylvania USA; ^3^ Center for Systems Neuroscience Boston University Boston Massachusetts USA

**Keywords:** analgesic tolerance, circadian genes, fentanyl, NPAS2, opioid side‐effects, physical dependence, sex differences

## Abstract

Opioids like fentanyl remain the mainstay treatment for chronic pain. Unfortunately, opioid's high dependence liability has led to the current opioid crisis, in part, because of side‐effects that develop during long‐term use, including analgesic tolerance and physical dependence. Both tolerance and dependence to opioids may lead to escalation of required doses to achieve previous therapeutic efficacy. Additionally, altered sleep and circadian rhythms are common in people on opioid therapy. Opioids impact sleep and circadian rhythms, while disruptions to sleep and circadian rhythms likely mediate the effects of opioids. However, the mechanisms underlying these bidirectional relationships between circadian rhythms and opioids remain largely unknown. The circadian protein, neuronal PAS domain protein 2 (NPAS2), regulates circadian‐dependent gene transcription in structure of the central nervous system that modulate opioids and pain. Here, male and female wild‐type and NPAS2‐deficient (NPAS2−/−) mice were used to investigate the role of NPAS2 in fentanyl analgesia, tolerance, hyperalgesia and physical dependence. Overall, thermal pain thresholds, acute analgesia and tolerance to a fixed dose of fentanyl were largely similar between wild‐type and NPAS2−/− mice. However, female NPAS2−/− exhibited augmented analgesic tolerance and significantly more behavioral symptoms of physical dependence to fentanyl. Only male NPAS2−/− mice had increased fentanyl‐induced hypersensitivity, when compared with wild‐type males. Together, our findings suggest sex‐specific effects of NPAS2 signaling in the regulation of fentanyl‐induced tolerance, hyperalgesia and dependence.

## Graphical abstract

Deficiency of the circadian transcription factor NPAS2 leads to augmented physical dependence to fentanyl in female mice.

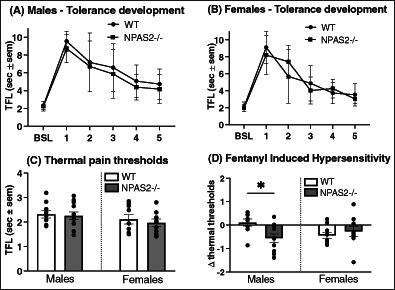

## 1 INTRODUCTION

Prescription opioids are potent analgesics used to treat pain. Extended use, often necessary to treat chronic pain, is a risk factor for developing tolerance (e.g., reduced effect of analgesia), physical dependence (i.e., withdrawal symptoms during periods of abstinence) and pain hypersensitivity (i.e., opioid‐induced increases in pain sensitivity),^1^, ^2^ and serves as a risk factor for opioid use disorder.^3^ Hallmarks of opioid dependence and opioid use disorder are profound disruptions to sleep and circadian rhythms that persist during abstinence and withdrawal.^4^, ^5^, ^6^, ^7^ In line with this, previous evidence suggests opioid tolerance, dependence and analgesia^8^, ^9^, ^10^, ^11^, ^12^ are modulated by sleep and circadian rhythms. Altered circadian rhythms and sleep disruptions are commonly observed in patients being treated with opioids.^13^ For example, sleep disturbances are a known indicator of opioid withdrawal symptoms in patients attempting to discontinue their opioid treatment.^14^ Sleep and circadian rhythm disruptions may also contribute to relapse risk in opioid abstinent patients.^15^, ^16^ However, our understanding of the molecular pathways that underlie the relationship between circadian rhythms and opioid tolerance and withdrawal is limited.

Nearly every cell in the body expresses the necessary machinery that controls circadian rhythms at the molecular level.^17^ The molecular clock is comprised of a series of interacting transcriptional and translational feedback loops. Transcription is driven by heterodimers of CLOCK (Circadian Locomotor Output Cycles Kaput Protein), or NPAS2 (neuronal PAS domain protein 2), with BMAL1 (brain muscle aryl nuclear translocase like‐1), that bind to enhancer elements in gene promoters. These heterodimers of circadian transcription factors (CLOCK or NPAS2 and BMAL1) drive the transcription of circadian genes, *Cry1,2* (*Cryptochrome*) and *Per1,2* (*Period*). Accumulation of CRYs and PERs in the cytoplasm over 24‐h initiate the formation of dimers that eventually translocate to the nucleus to inhibit their own transcription via interactions with BMAL1 heterodimers.^18^ Molecular clocks are critical to the function of most peripheral organs and many regions in the brain.^19^

In peripheral tissues and the central nervous system, the molecular clock modulates the circadian expression of endogenous opioid peptides and opioid receptors.^20^, ^21^, ^22^, ^23^ Opioids also impact the expression of circadian genes in the brain. For example, acute and chronic administration of opioids alters circadian genes in the hypothalamus,^24^, ^25^, ^26^ while withdrawal from opioids alters the rhythmic expression of circadian genes in the midbrain and striatum.^26^, ^27^, ^28^ Recent findings show circadian genes, including *Per1* and *Per2*, regulate opioid reward,^29^ tolerance and withdrawal.^30^ In a tissue‐dependent manner, *Per1* and *Per2* transcription are driven by BMAL1 dimerization with CLOCK or NPAS2.^31^, ^32^, ^33^ Notably, the circadian transcription factor NPAS2 seems to be predominantly expressed in the central nervous system^34^ and enriched in major neural substrates of opioid‐induced tolerance,^35^ dependence,^36^, ^37^, ^38^ and hyperalgesia,^39^ including the spinal cord,^34^ primary sensory neurons,^40^ and within the subregions of the striatum, including the nucleus accumbens (NAc).^41^ However, to our knowledge, the involvement of NPAS2 signaling in the emergence of opioid side‐effects has not yet been studied.

In the present study, we investigated the role of NPAS2 in opioid tolerance, dependence and analgesia, using the synthetic opioid, fentanyl. Fentanyl is a widely prescribed opioid with high potential for dependence, found in most drug overdose deaths in the United States.^3^ Thus, further investigation into the potential pathways mediating the development of fentanyl‐induced tolerance and dependence is imperative, as new therapeutic approaches are designed to improve opioid treatments and mitigate secondary effects of opioids. To explore the potential relationship between NPAS2 and opioids, we assayed behavioral phenotypes of fentanyl‐induced tolerance, dependence and hyperalgesia in NPAS2 deficient male and female mice (NPAS2−/−). These transgenic mice carry a LacZ reporter that replaces exon 2 of the *Npas2* gene and effectively deletes the basic Helix–loop–Helix (bHLH) domain required for NPAS2 to directly bind DNA. The lack of the bHLH domain impedes NPAS2‐dependent transcription.^32^ Therefore, NPAS2‐deficient (NPAS2−/−) mice retain the expression of the NPAS2 protein, while lacking the ability to bind DNA and drive transcription dependent on NPAS2. Overall, our findings reveal sex‐specific effects of NPAS2 deficiency on thermal nociceptive thresholds, acute anti‐nociception and development of tolerance to fentanyl, as well as behavioral markers of physical dependence and pain hypersensitivity.

## 2 MATERIALS AND METHODS

### 2.1 Animals

Adult male and female NPAS2‐deficient (NPAS2−/−) mice and their respective wild‐type (WT) littermates were used (aged 7–14 weeks).^32^ Original breeders were generously provided by Dr. David Weaver (University of Massachusetts Medical School). NPAS2−/− mice have a LacZ‐Neo fusion gene inserted into exon 2, replacing exon 2 that encodes the bHLH domain, rendering NPAS2 unable to directly bind DNA to regulate NPAS2‐dependent transcription. Mice were maintained on C57BL/6J background (The Jackson Laboratory, 000664; backcrossed to at least N10). Mice were group housed (2–4 mice per cage) and maintained under 12 h:12 h standard light–dark cycle (Lights on at 0700 h, Zeitgeber time [ZT] 0 and lights off at 1900 h, ZT12) with ad libitum access to food and water. Unless otherwise indicated, all injections and behavioral procedures were started at ~ZT2 (0900 h). Experimental procedures were approved by the Institutional Animal Care and Use Committee at the University of Pittsburgh School of Medicine.

### 2.2 Drugs

Fentanyl hydrochloride (NIH NIDA, Bethesda, MD) and Naloxone (Sigma, St Louis, MO) were dissolved in sterile saline vehicle (0.9%) under a biosafety cabinet. Fentanyl was administered intraperitoneally (i.p., 10 ml/kg, vol/wt).

### 2.3 General procedures and experimental timeline

**FIGURE 1:**
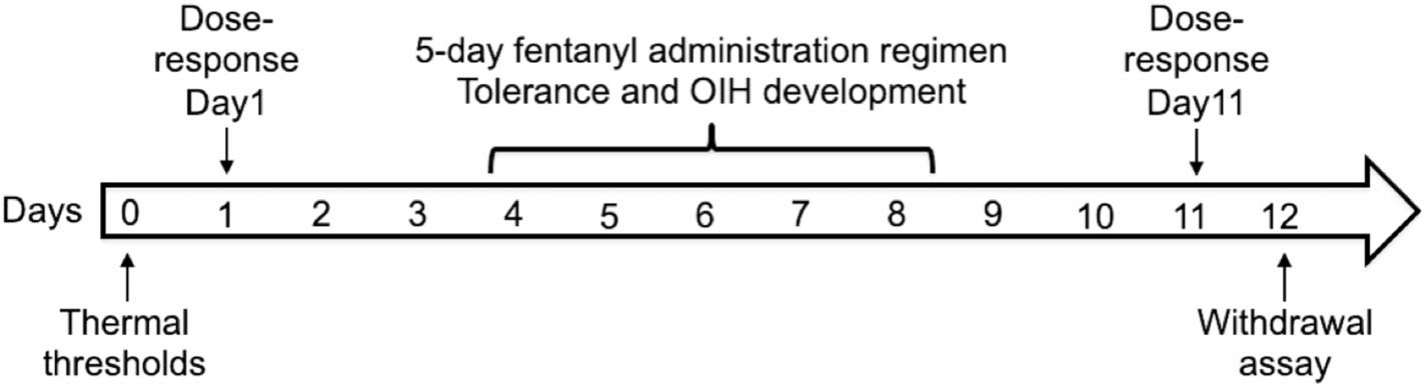
Experimental timeline. All animals underwent the same procedures. On day 0, animals were tested for thermal nociceptive thresholds (baselines) with the tail flick (TFL) assay. On day 1, a first dose–response of the fentanyl analgesic effect on TFL was performed. The 5‐day fentanyl administration regimen involving two fentanyl i.p. injections per day was conducted from day 4 to day 8. Tolerance and OIH development were evaluated simultaneously. On day 11, a second fentanyl dose–response was conducted. This series of experiments concluded with the naloxone‐precipitated withdrawal assay on day 12

Throughout the experiments, experimenters were blind to both the treatment and genotype groups. Prior to each behavioral assay, mice were habituated to experimental rooms and gently handled by experimenters for at least 1 h per day for three consecutive days. We used 9–11 mice per genotype per sex. The experimental timeline is described in Figure 1. On day 0, baseline thermal thresholds were measured from tail flick latencies (TFL) averaged over three trials per mouse. On day 1, mice underwent a first dose–response procedure with fentanyl (i.p.). TFLs were measured 15 min after fentanyl administration. The fentanyl dose was then doubled every 20 min, and tested again until TFLs reached the threshold of 10 s. The following fentanyl doses were administered on day 1: 10, 20, 40, 80, 160 and 320 μg/kg. Between days 4 and 8, mice were tested for the development of tolerance and hyperalgesia. Mice were administrated fentanyl (320 μg/kg, i.p.) twice daily at ~ZT2 (0900 h) and ~ZT9 (1600 h). Testing of TFL was performed before and 15 min after the ZT2 administration to test development of hyperalgesia and tolerance, respectively. On day 11, a second dose–response was conducted using the following fentanyl doses: 160, 320, 640 and 1280 μg/kg. Finally, on day 12, mice underwent naloxone‐precipitated withdrawal, as described below.

### 2.4 Tail‐immersion test for fentanyl‐induced analgesia and tolerance

The tail‐immersion behavioral assay was used to measure TFL, and assess thresholds of thermal nociception, analgesia and tolerance to fentanyl. A container filled with water maintained at 50 ± 0.5°C using a thermoregulated water bath. Part of the mouse's tail was immersed in the water and the TFL was recorded with a maximum immersion limit of 10 s to avoid tissue damage. After testing, mice were immediately returned to their home cage. TFL measurements were repeated 2–3 times per mouse at 2‐min intervals.

### 2.5 Naloxone‐precipitated fentanyl withdrawal

Following the development of physical dependence to fentanyl, behavioral signs of withdrawal from chronic fentanyl were evaluated using the opioid receptor antagonist, naloxone. Mice were administered fentanyl (320 μg/kg, i.p.), then naloxone (10 mg/kg, i.p.) ~15 min later. Withdrawal behaviors were recorded for 10 min in a novel environment composed of a transparent Plexiglas chamber with bedding. Recorded behaviors included escape jumps, wet‐dog shakes, paw‐shakes/grooming, teeth chattering. Presence of diarrhea was also recorded. Behaviors were compiled to calculate cumulative withdrawal scores as follows: Jumps: 1–10 = 2, 11–20 = 4, 21–30 = 6, 31–40 = 8, 41–50 = 10 and so forth; wet dog shakes: 1–2 = 2, 3–10 = 4, 10 and more = 6; and presence of teeth chattering, paw shakes and diarrhea were scored a 2.

### 2.6 Statistical analyses

A combination of statistical analyses was used for behavioral assays. Tolerance measures were analyzed using two‐way analysis of variance (ANOVA) (Time and Treatment) followed by Tukey's multiple comparison tests. Thermal nociceptive thresholds, EC50s and withdrawal behaviors were analyzed using one‐way ANOVA, followed by Tukey's post‐hoc multiple comparison tests, when appropriate. Dose–response curves were generated using a non‐linear regression of log‐transformed values compared with normalized values from 0 to 100. Rightward shifts in the dose–response curves between mouse genotypes were compared by calculating the ratio between EC50 obtained on day 11 versus EC50 obtained on day 1. Unpaired *t* test comparisons were performed to determine difference of rightward shift between NPAS2−/− and WT mice of the same sex. Data were analyzed using GraphPad 9.0 and considered statistically significant if *p* ≤ 0.05.

## 3 RESULTS

### 3.1 Impact of NPAS2 deficiency on thermal thresholds, fentanyl acute analgesia, fentanyl‐mediated tolerance and hypersensitivity

**FIGURE 2:**
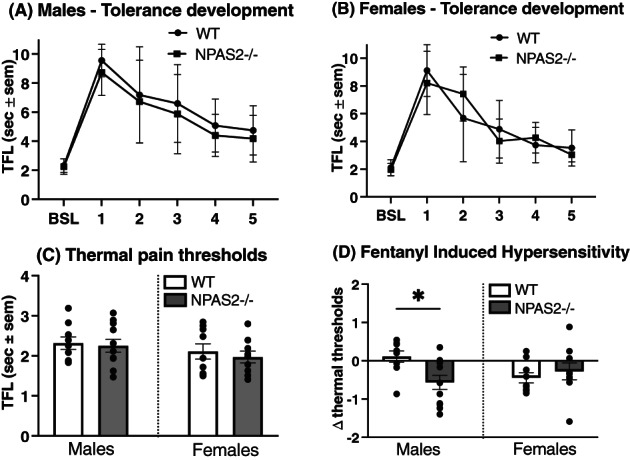
Thermal nociceptive thresholds and fentanyl analgesic tolerance in male and female wild‐type and NPAS2‐deficient mice. Development of tolerance to a fixed dose of fentanyl (320 μg/kg) administered twice a day for 5 days was assessed my measuring tail flick latencies (TFLs), daily in males (A), and females (B). Two‐way analysis of variances (ANOVAs), males, *N* = 9–11, interaction: *F*_5,90_ = 0.1601, *p* = 0.9764, days: *F*_3.058,55.04_ = 48.38, *p* < 0.0001, Treatment: *F*_1,18_ = 0.7066, *p* < 0.0001; females, *N* = 11, Interaction: *F*_5,80_ = 2.233, *p* = 0.0590, days: *F*_3.211,51.37_ = 50.40, *p* < 0.0001, Treatment: *F*_1,16_ = 0.001806, *p* < 0.9666. (C) Baseline thermal nociceptive thresholds measured prior to the beginning of fentanyl injections. One‐way ANOVA, *N* = 9–11, *F*_3,34_ = 0.8418, *p* = 0.4805. (D) Comparison of baseline thermal threshold measured on day 0, to threshold measured on day 5 before the fentanyl injection. Data represented as delta of TFL values measured on Day 5–Day 0, one‐way ANOVA, *N* = 9–11, *F*_3,34_ = 2.765, *p* = 0.0568. Data represented as mean ± SEM. BSL, baseline; TFL, tail flick latency

The impact of NPAS2 deficiency on thermal thresholds and development of tolerance and hypersensitivity with chronic fentanyl was evaluated by measuring TFLs in NPAS2−/− and WT mice before and after 5 days of fentanyl administration (Figure 1). The acute analgesic effect of fentanyl measured on day 1 was similar between NPAS2−/− and WT male (Figure 2A) and female (Figure 2B) mice. Additionally, all groups had a similar progressive reduction in TFLs over time without a significant difference between genotypes in males (Figure 2A, two‐way ANOVA, interaction: *F*_5,90_ = 0.1601, *p* = 0.9764, days: *F*_3.058,55.04_ = 48.38, *p* < 0.0001, treatment: *F*_1,18_ = 0.7066, *p* < 0.0001) and females (Figure 2B, two‐way ANOVA, interaction: *F*_5,80_ = 2.233, *p* = 0.0590, days: *F*_3.211,51.37_ = 50.40, *p* < 0.0001, treatment: *F*_1,16_ = 0.001806, *p* < 0.9666). Similarly, baseline TFLs measured in opioid naïve animals prior to fentanyl administration were largely similar between NPAS2−/− and WT male and female mice (Figure 2C), suggesting that NPAS2 has minimal impact on thermal nociceptive thresholds in opioid naïve mice (one‐way ANOVA, *F*_3,34_ = 0.8418, *p* = 0.4805). However, thermal thresholds prior to fentanyl administration on day 1 compared with day 5 revealed NPAS2−/− male mice developed thermal hypersensitivity, an effect absent in WT male mice. Conversely, both NPAS2−/− and WT females developed thermal hypersensitivity (Figure 2D, day 5–day 1 TFLs, *F*_3,34_ = 2.765, *p* = 0.0568). Together, these findings support the involvement of NPAS2 in hyperalgesia development in a sex‐specific manner.

### 3.2 Impact of NPAS2 deficiency on sex‐specific changes in fentanyl potency

**FIGURE 3:**
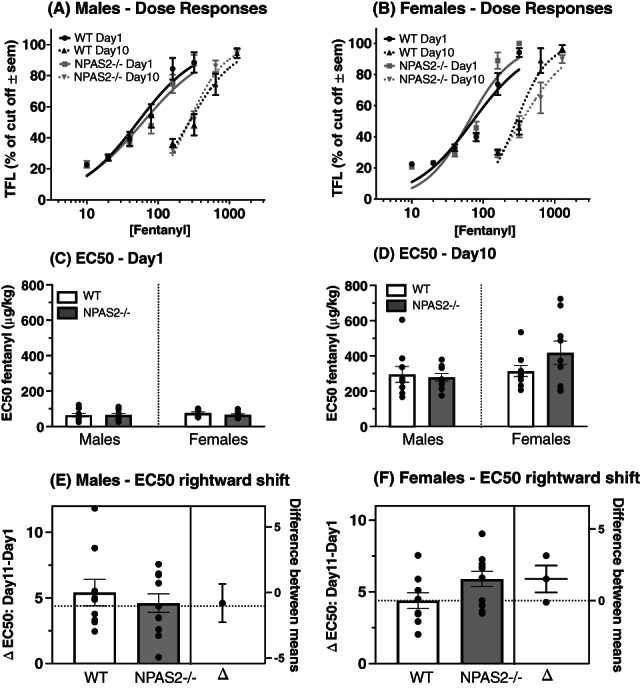
NPAS2‐deficiency alters fentanyl potency in female mice. Fentanyl dose–response curves in males (A), and females (B) measured before (day 1) and after (day 11) induction of tolerance with fentanyl. Doses administered ranged from 10 to 320 μg/kg for during the pre‐tolerance test, and from 160 to 1280 μg/kg during the post‐tolerance test. Data represented as normalized log[fentanyl] and normalized from 0 to 100. *N* = 9–11, Non‐linear fit, Best‐fit values for EC50 values calculations: Males: WT pre‐tolerance: 51.75 μg/kg, WT post‐tolerance: 273.4 μg/kg, 5.1‐fold rightward shift; NPAS−/− pre‐tolerance: 61.25 μg/kg, NPAS−/− post‐tolerance: 269.6 μg/kg, 4.4‐fold rightward shift. Females: WT pre‐tolerance: 71.24 μg/kg, WT post‐tolerance: 291.0 μg/kg, 4.08‐fold rightward shift; NPAS−/− pre‐tolerance: 63.01 μg/kg, NPAS−/− post‐tolerance: 346.0 μg/kg, 5.5‐fold rightward shift. (C) Day 1 EC50s comparisons, *N* = 9–10, one‐way analysis of variance (ANOVA), *F*_3,33_ = 0.4501, *p* = 0.7189. (D) Day 11 EC50s comparisons, *N* = 9–10, one‐way ANOVA, *F*_3,33_ = 2.056, *p* = 0.1251. (E) Comparison of degree of rightward shift of EC50s between day 1 and day 11 (EC50 day 1–EC50 day 11) degree between WT and NPAS2−/− males, *N* = 9–11, two‐tailed *t* test, *t* = 0.6714, df = 18, *p* = 0.5105. (F) Comparison of degree of rightward shift of EC50s between day 1 and day 11 (EC50 day 1–EC50 day 11) degree between WT and NPAS2−/− females, *N* = 9, two‐tailed *t* test, *t* = 2.096, df = 16, *p* = 0.0523. (C–D) Data represented as mean ± SEM. EC50, effective dose 50; TFL, tail flick latency

To further test the impact of NPAS2 deficiency on fentanyl analgesic potency, we performed two fentanyl dose–response procedures on male and female NPAS2−/− and WT mice. The first dose–response was conducted on day 1 when mice were naïve to opioids (Figure 3A), and the second dose–response was conducted on day 11, after mice had received the tolerance‐inducing 5‐day fentanyl administration regimen (Figure 3B). Dose‐responses performed on day 11, revealed a rightward shift of the fentanyl dose–response curves in all groups, as compared with dose–responses measured on day 1 (Males, Figure 3A, Females, Figure 3B). Therefore, our regimen induced a robust state of analgesic tolerance in all mice. Based on best‐fit values of these curves, we calculated effective concentration 50 (EC50) values, which represent the concentration of fentanyl that gives half‐maximal analgesia (EC50 day 1 values: WT males: 51.75 μg/kg; NPAS2−/− males: 61.25 μg/kg; WT females: 71.24 μg/kg; and NPAS2−/− females: 63.01 μg/kg. EC50 day 11 values: WT males: 273.4 μg/kg, NPAS2−/− males: 269.6 μg/kg, WT females: 291.0 μg/kg and NPAS2 females: 346.0 μg/kg). For EC50 values, no significant differences between NPAS2−/− and WT mice were found in pre‐tolerance fentanyl potency on day 1 (Figure 3C, one‐way ANOVA, *F*_3,33_ = 0.4501, *p* = 0.7189). On day 10, EC50 values were also similar between genotypes and among both sexes (Figure 3D, one‐way ANOVA, *F*_3,33_ = 2.056, *p* = 0.1251). Finally, calculation of the rightward shift factor between naïve (day 1) and tolerant (day 11) mice (EC50 day 1–EC50 day 11), revealed that degree of rightward shift of curves in WT and NPAS2−/− males were similar (Figure 3E, two‐tailed *t* test, *t* = 0.6714, df = 18, *p* = 0.5105). Conversely, comparison of shift factors in female mice revealed a greater shift in NPAS2−/− females (6.069 rightward shift average), than in WT females (4.382 rightward shift average) that was close to statistical significance (Figure 3F, two‐tailed *t* test, *t* = 2.096, df = 16, *p* = 0.0523). Overall, our findings point toward an impact of NPAS2 deficiency on fentanyl potency in female, but not male mice.

### 3.3 Impact of NPAS2 deficiency on naloxone‐precipitated withdrawal responses in fentanyl‐dependent mice

**FIGURE 4:**
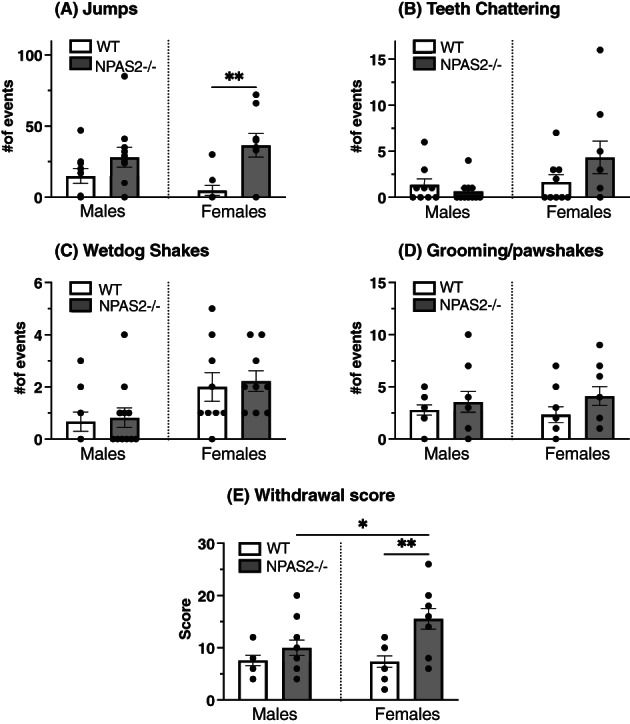
NPAS2‐deficiency potentiates physical dependence behaviors to fentanyl in female mice. Naloxone‐precipitated withdrawal behaviors in NPAS2−/− and WT littermate mice administered with a challenge dose of fentanyl (320 μg/kg, i.p.). (A) Total number of jumps, *N* = 9–11, one‐way analysis of variance (ANOVA), *F*_3,34_ = 4.646, *p* = 0.0079. (B) Total number of wet dog shake episodes, *N* = 9–11, one‐way ANOVA, *F*_3,34_ = 3.425, *p* = 0.0279. (C) Total number of teeth chattering episodes, *N* = 9–11, one‐way ANOVA, *F*_3,34_ = 2.624, *p* = 0.0663. (D) Total number of paw shakes/grooming episodes, *N* = 9–11, one‐way ANOVA, *F*_3,34_ = 0.8478, *p* = 0.4774. (E) Withdrawal score, *N* = 9–11, one‐way ANOVA, *F*_3,34_ = 6.543, *p* = 0.0013. Data represented as mean ± SEM. Tukey's multiple comparisons tests, *p < 0.05, **p < 0.01, ***p < 0.001

Following the post‐tolerance dose–response regimen of fentanyl, mice received a fentanyl challenge, followed by naloxone, to precipitate withdrawal and induce dependence behaviors. Overall, female NPAS2−/− mice displayed significantly more withdrawal behaviors than NPAS2−/− and WT male mice. Fentanyl naloxone precipitated withdrawal led to more jumps in NPAS2−/− females compared with WT females (Figure 4A, one‐way ANOVA, *F*_3,34_ = 4.646, *p* = 0.0079), while these behaviors were similar between genotypes in males. The number of wet‐dog shakes were overall unchanged in NPAS2−/− mice (Figure 4B), although female mice had significantly more wet‐dog shakes than males, regardless of genotype (Figure 4B, one‐way ANOVA, *F*_3,34_ = 3.425, *p* = 0.0279). In addition, teeth‐chattering episodes were more frequent in NPAS−/− mice as compared with WT mice, but this effect was not significant (Figure 4C, one‐way ANOVA, *F*_3,34_ = 2.624, *p* = 0.0663). Finally, no difference was observed in the number of grooming and paw shaking episodes across groups (Figure 4D, one‐way ANOVA, *F*_3,34_ = 0.8478, *p* = 0.4774). Global withdrawal scores, which involved the number of jumps, along with the number of teeth‐chattering, wet dog shakes, grooming/paw shakes episodes, showed that NPAS2−/− females displayed significantly more withdrawal behaviors as compared WT female mice and male mice from both genotypes (Figure 4E, one‐way ANOVA, *F*_3,34_ = 6.543, *p* = 0.0013.). Together, these results support a role of NPAS2 in the development and exhibition of physical dependence primarily in female than in male mice.

## 4 DISCUSSION

Long‐term opioid use for the treatment of chronic pain is hampered by analgesic tolerance,^42^ physical dependence,^43^ and disruptions of sleep and circadian rhythms.^4^, ^5^, ^6^, ^7^ Previous studies implicated circadian regulation of pain, tolerance and physical dependence,^11^, ^12^ suggesting bidirectional interactions between circadian rhythms and opioids.^9^ NPAS2 is a circadian gene enriched in spinal cord and brain regions involved in pain and opioids, and previously shown to be involved in psychostimulant reward.^44^, ^45^ However, the role of NPAS2 in opioid tolerance and dependence had yet to be established. To address this, we used NPAS2‐deficient mice to investigate the involvement of NPAS2 on fentanyl‐mediated tolerance, hypersensitivity and physical dependence. Overall, we found that thermal pain thresholds, acute fentanyl analgesia and fentanyl tolerance development were unchanged in male and female NPAS2−/− mice. Interestingly, NPAS2 deficiency led to a decrease in fentanyl potency after tolerance developed and led to markedly more symptoms of physical dependence only in female mice. Conversely, NPAS2 deficiency was associated with increased fentanyl‐induced hypersensitivity in male compared with female mice.

Our results are consistent with prior studies which also evaluated the impact of genetic deletion of circadian genes on pain and opioids.^29^, ^30^ In these studies, mice with global deletion of *mPer1* (mPer1‐KO)^29^ or *mPer2* (mPer2‐KO)^30^ genes showed no change in thermal pain thresholds and acute analgesia. Similarly, NPAS2−/− mice exhibited no measurable difference in thermal pain thresholds and acute analgesia, suggesting these circadian genes may not be directly involved in these behaviors. Conversely, *mPer1* and *mPer2* clock genes were differentially involved in tolerance and dependence, as mPer2‐KO promoted morphine tolerance and mitigated physical dependence,^30^ while mPer1‐KO did not affect tolerance or physical dependence.^29^ In our study, which included both males and females, NPAS2−/− mice did show altered tolerance development to a fixed dose of fentanyl across both sexes. In addition, we also evaluated fentanyl potency, by conducting dose–response tests before and after tolerance development. Strikingly, NPAS2−/− mice developed higher analgesic tolerance compared with controls, an effect only observed in females. These behaviors are thought to be mediated by different mu‐opioid receptor (MOR) signaling mechanisms, possibly in a cell type‐specific and/or region‐specific manner.^46^ Importantly, tolerance can develop to all MOR‐mediated behaviors, although at different rates.^1^, ^47^ The apparent dichotomy between the decreased analgesic potency of fentanyl while withdrawal behaviors were increased in NPAS2−/− females, could be explained by either a distinct involvement of NPAS2 in analgesia and physical dependence, or perhaps pronounced differential development of tolerance to analgesia and to physical dependence in these mice. Nevertheless, these data could suggest that circadian genes are differentially involved in tolerance, with *mPer2* being involved in its development, *Npas2* in its expression and *mPer1* likely not involved. However, caveats of studies investigating the roles of *Per* genes include the absence of testing opioid potency in tolerant animals with a dose–response assay, along with these studies including only male mice. Because *Npas2* and *Per* genes are both expressed in structures involved in tolerance such as the spinal cord^34^, ^48^ or the NAc,^49^ and NPAS2 can directly regulate the transcription of *Per* genes, involvement of each of these circadian genes in tolerance remains a possibility.

Sex specific effects of NPAS2 deficiency were also observed with physical dependence symptoms. NPAS2−/− females showed more withdrawal behaviors than controls, also more than NPAS2−/− males. Conversely, when testing fentanyl‐induced hypersensitivity, a symptom also emerging during opioid withdrawal, NPAS2−/− females were like WT females, while NPAS2−/− males had more pronounced hypersensitivity than WT males. Together, these results suggest that tolerance, physical dependence and opioid‐induced hypersensitivity behaviors could be modulated by NPAS2 signaling in a sex specific manner.

Involvement of NPAS2 in these behaviors could be supported by the fact that NPAS2 expression is enriched in the NAc.^32^, ^41^ The NAc is involved in tolerance,^49^, ^50^ physical dependence,^37^, ^51^, ^52^ and opioid‐induced hyperalgesia.^12^ NPAS2 modulates dopaminergic and glutamatergic neurotransmission in the striatum,^53^ both of which are altered during opioid tolerance^49^, ^50^ and withdrawal.^54^, ^55^, ^56^ Interestingly, changes in the expression of circadian genes in the NAc were shown to occur in rodents with opioid‐induced hyperalgesia during a state of withdrawal.^12^ Thus, together with our current findings, NPAS2 may modulate opioid‐related behaviors and involve dopaminergic and glutamatergic signaling in the NAc. In addition, NPAS2 may also play a role in peripheral tissues^57^, ^58^ and NPAS2 is expressed in primary sensory neurons,^40^ which are known to be essential in opioid tolerance and hyperalgesia behaviors.^39^ Ongoing and future studies are exploring possible involvement of NPAS2 in the NAc dopaminergic and glutamatergic neurotransmission in opioid tolerance and dependence.

Our results illustrate the importance of examining interactions between sex, opioid behaviors and circadian genes. This is also supported by several previous studies, which examined whether sex could have a differential impact on opioid behaviors. Analgesic effect of opioids was shown to be variable depending on sex, with a higher and longer‐lasting effect in male than female rodents,^59^, ^60^, ^61^ although, other studies did not find a sex difference in opioid analgesia.^62^, ^63^ In addition, sexual dimorphism in opioid tolerance has not been extensively studied in rodents, yet the studies that have, report higher tolerance in males than females,^64^ with tolerance developing faster in females than males.^65^, ^66^ However, these findings have not been supported by other studies.^67^, ^68^, ^69^ Finally, sexual dimorphism in opioid dependence‐mediated withdrawal behaviors were also reported in rodent studies, with overall more dependence in WT males than WT female rats.^64^, ^70^ Inconsistent findings between men and women have also been reported in humans.^71^, ^72^, ^73^, ^74^ In our current study, we did not observe sex‐related differences in opioid analgesia, tolerance development and expression, dependence and hyperalgesia, between WT male and female mice. Collectively, our data illustrates the lack of consensus on the impact of sex on opioid behaviors, and thus requires further investigation.

Nevertheless, in our study, we specifically examined intersectional consequences of NPAS2 deficiency and sex on opioid analgesia, tolerance and dependence behaviors. Interestingly, NPAS2−/− females developed markedly more physical dependence behaviors and marginally more profound tolerance than female WT littermates, which was not observed in males. However, NPAS2 deficiency had no consequences on hyperalgesia development in females while it exacerbated that symptom in males. Thus, our data indicates that sex differences in our study are related to interacting effects between NPAS2 deficiency and sex. This could be explained by the fact that sex differences are also known to exist in circadian rhythms^75^, ^76^ and in circadian genes rhythmicity between males and females in brains of humans^77^ and of rodents.^78^

Our results are also consistent with prior studies which examined intersectional consequences between sex and circadian genes such as *Clock* or *Npas2*.^44^, ^79^ Interestingly, *Npas2* deletion had higher impact on cocaine reward and self‐administration behaviors in female mice.^44^ More profound consequences on females than males could be explained by levels of circulating hormones, as sex differences in cocaine self‐administration were abolished in ovariectomized females.^44^ This was consistent with the fact that circulating estrogens were shown to be essential in orchestrating rhythms of circadian genes in the SCN.^80^ In addition, estrogen signaling has been shown to influence opioid tolerance and dependence behaviors.^81^ Thus, female circulating hormones could also be involved in the sexually dimorphic consequences of NPAS2 deficiency on opioid tolerance and dependence. Further studies examining the mechanisms of interaction between NPAS2, opioids sexual hormones are now warranted.

Overall, our study and prior studies examining interactions between circadian genes and opioid‐related behaviors, show a differential involvement of *Npas2 and Per* genes.^29^, ^30^ However, *Npas2* and *Per* genes bi‐directionally regulate their expression levels, leading to variations in expression that follow circadian rhythmicity.^33^ Importantly, rhythmic expression of these circadian genes follow different circadian phases.^82^, ^83^ Therefore, suggesting that involvement in behavior of these genes could vary at different times of day. In our study, we examined the role of NPAS2 deficiency at a similar time of day (ZT2) as the mPer1‐KO and mPer2‐KO studies (ZT3‐5).^29^, ^30^ Whether these mutations could impact opioid‐related behaviors via alterations of the circadian clock remains unknown.^31^, ^32^, ^45^ However, other behaviors involving NAc circuitry, like feeding, and food or cocaine self‐administration, were shown to be differentially altered at varying times of day in NPAS2−/− mice.^44^, ^45^ Therefore, ongoing studies are now also evaluating whether impact of NPAS2‐defficiency on opioid tolerance and physical dependence vary at different times of day.

While prior studies used morphine to evaluate the impact of *mPer1* and *mPer2* deletion, we used fentanyl to evaluate impact of NPAS2 deficiency. Although both opioids modulate analgesia via MORs,^84^, ^85^ they can activate different MOR downstream signaling pathways.^86^, ^87^, ^88^ Therefore, fentanyl and morphine may recruit different circadian genes and downstream signaling pathways involving MOR.

In conclusion, our study provides evidence for a differential role of NPAS2 signaling in fentanyl mediated behaviors, with high impact on physical dependence and marginal effect on tolerance. Importantly, NPAS2 deficiency modulated these behaviors in a sexually dimorphic manner, with female mice more profoundly affected than males. Identification of NPAS2‐controlled genes and signaling pathways that modulate opioid behaviors and that may interact with substrates of opioid tolerance and dependence, could provide better insight on understanding the impact of clock genes and circadian rhythmicity on the chronic use of prescription opioids in patients.

## Data Availability

The data that support the findings of this study are available from the corresponding author upon reasonable request.
